# MPOWER: The Impact of a Purpose Program on Adolescents’ Intrinsic and Extrinsic Motivations

**DOI:** 10.3389/fpsyg.2021.761580

**Published:** 2021-12-16

**Authors:** Jonathan A. Sepulveda, Brenna Lincoln, Belle Liang, Timothy Klein, Allison E. White, Nancy Hill, John Perella

**Affiliations:** ^1^Counseling Psychology, Felician University, Lodi, NJ, United States; ^2^Counseling and Developmental Psychology, Boston College, Chestnut Hill, MA, United States; ^3^Boston College, Chestnut Hill, MA, United States; ^4^Massachusetts General Hospital, Boston, MA, United States; ^5^Graduate School of Education, Harvard University, Cambridge, MA, United States; ^6^Revere High School, Revere, MA, United States

**Keywords:** adolescents, self-efficacy, goal orientation, program evaluation, purpose, academic performance

## Abstract

Purpose has been defined as an active engagement toward goals that are meaningful to the self (i.e., personal meaningfulness) and contribute to the world beyond the self (BTS). These BTS contributions may reflect the intention to meet a wide range of needs from family financial needs to more macro-level concerns, including social injustices. This study investigates the efficacy of a school-based program called MPOWER expressly designed by the authors to cultivate the BTS aspect of purpose. Previous research suggests that the BTS aspect of purpose has beneficial effects on school engagement, goal-setting abilities and orientations, and ultimately school performance. Ninety-four students participated in this study that utilized a randomized, pre-test-post-test between-subjects design to evaluate MPOWER (52 in MPOWER and 42 in the control group). The ANCOVA results indicated a significant increase in the BTS aspect of purpose among program participants, compared to controls. Moreover, participants had higher post-test levels of general self-efficacy and grade point averages, and decreased performance-approach (e.g., playing to be the best, comparing self to others) and performance-avoidance (e.g., avoiding risks of failure, fear of social consequences) goal orientations. Findings can be used to design programs that aim to cultivate students’ intentions to contribute to the world beyond themselves, as well as associated personal benefits (i.e., goal orientations, self-efficacy, academic performance).

## Introduction

Purpose has been defined as a long-term goal or aim that is both personally meaningful and contributes to the world beyond-the-self (Damon, 2009). Within this definition, aims that are pursued solely for the benefit of the self (e.g., I want to make a lot of money) are not as meaningful as aims that benefit the self and greater society (e.g., I want to make a lot of money so that I can help build homes for poor families). As it turns out, this beyond-the-self (BTS) intention to do good in the world is also beneficial for the doers. The BTS aspect of purpose may play a particularly important role in the lives of marginalized adolescents for whom purpose may increase resiliency, community connection, and the desire to challenge systemic oppression that affects their marginalized group (Sumner et al., 2018). Moreover, purpose has been associated with increased academic engagement (Damon, 2009; Liang et al., 2016, 2017) and grade-point average (Martin and Martin, 1977). As important as having purpose may be for adolescent outcomes, there are few published studies describing and evaluating programs designed to cultivate purpose.

MPOWER is a purpose program that reflects key influences on the development of purpose, called the 4 P’s of purpose: (1) *people* who provide the necessary support; (2) *passion* or one’s long-standing interests; (3) *propensity* or one’s skills and strengths related to their purpose, and (4) *prosocial benefits* or an intention to contribute to others beyond the self (Liang et al., 2016, 2017). That is, the program works through key *people* or relationships who help adolescents identify and cultivate their *passion, propensity*, and desired *prosocial benefits* as these relate to their’ long-term aspirations or purpose.

In order to foster the development of the BTS aspect of purpose, MPOWER helped participants reflect on prosocial benefits and contributions. Participants became engaged in activities that encouraged them to think about the impact their purpose may have on others. The BTS aspect of purpose is highlighted because when adolescents connect their actions to a positive impact in the world, they view their academic efforts as worthy and relevant (Yeager et al., 2014). Additionally, the MPOWER curriculum was designed to raise adolescents’ self-efficacy in order to embolden them to pursue experiences associated with their purpose and subsequently build on their existing interests, strengths, and skills. MPOWER also attempts to foster a goal orientation characterized by an intention to increase personal competence and knowledge (i.e., mastery) vs. striving for social acceptance (i.e., performance-approach) in its participants. In other words, MPOWER aims to help participants develop intrinsic motivations (i.e., personal interest), rather than the desire to meet the expectations of others (i.e., approval from others). When individuals are motivated by this healthier aspiration to master skills that are intrinsically interesting in contrast to being accepted by others, they tend to see their daily activities (e.g., school activities, classroom assignments) as more related to their purpose.

The aim of this paper was to address this gap by evaluating the efficacy of MPOWER and the resulting influence that the adolescents’ increased sense of purpose had on their academic performance and engagement in a sample of high school students. In particular, we examined the outcomes of MPOWER on GPA, as well as the impact it has on both aspects of purpose (i.e., personal meaningfulness, BTS), self-efficacy and goal-orientation.

### Purpose and Academic Engagement

Adolescents who have a purpose are more academically engaged than their peers (Hill et al., 2018; Klein et al., 2019). Purposeful adolescents compared to their counterparts get better grades (Adelabu, 2008), perform better on intelligence tests (Minehan et al., 2000), hold more positive academic self-identities (Dukes and Lorch, 1989), and are more academically motivated than are their peers (Nurmi, 1991). When an adolescent has a meaningful purpose, not only does it cultivate a future orientation, it guides and motivates present behavior toward achieving future-oriented goal(s) (Damon et al., 2003). For example, adolescents who were motivated by future goals exhibited better studying behaviors (Horstmanshof and Zimitat, 2007) and greater intrinsic interest in academics (Brown and Jones, 2004).

The BTS aspect of purpose highlights an intention to pursue aims that transcend the self and impact other people, the greater society, or the world. Adolescents who are committed to a purpose(s) that would impact others, possibly their own families, communities, and/or social groups, may be more likely to persist despite obstacles and adversity (Bronk and Finch, 2010; Sumner et al., 2018). Adolescents who experience marginalization may commit to a purpose that includes a prosocial (i.e., BTS) goal of improving the issues faced by their social group through civic or political action (Malin et al., 2015; Godfrey and Cherng, 2016). Additionally, a prosocial, self-transcendent purpose was associated with academic self-regulation and increased deeper learning behaviors (Yeager et al., 2014).

Two centrally notable variables that seem to work alongside purpose to encourage academic engagement and performance are goal orientation (Bronk et al., 2009) and self-efficacy (DeWitz et al., 2009). For example, research suggests that purposeful adolescents are more likely to be motivated toward intrinsic goals rather than extrinsic goals (Damon et al., 2003; Damon, 2009). Moreover, adolescents who are motivated by intrinsic goals tend to have a higher sense of self-esteem and self-concept (Middleton and Midgley, 1997; Turner and Patrick, 2004).

### Purpose and Goal Orientation

Researchers have drawn connections between the development of purpose and intrinsic vs. extrinsic motivation (Liang et al., 2016). Adolescents are bombarded by societal values that prioritize extrinsic goals (e.g., pushing ahead of others and self-focused success for the sake of power, prestige, and money). Yet, some adolescents demonstrate more intrinsic motivations that are associated with purpose. Purpose, by definition, is a life aspiration that is both personally meaningful and beneficial to society (Damon et al., 2003).

Understanding how adolescents develop these intrinsic vs. extrinsic orientations toward their long-term goals and aspirations are a key to understanding how to foster the BTS aspect of purpose (Liang et al., 2016). Dweck (1986) developed a goal orientation framework that explains students’ approaches to goals as falling into two main categories: (1) performance and (2) learning or mastery. A performance goal orientation is bound by social comparison and is characterized by a desire to gain positive judgments and/or avoid negative judgments (Dweck, 1986). This motivation differs from a genuine, intrinsic interest in the goal, and a desire to become more competent and knowledgeable (Pintrich, 2000).

Performance goal orientation includes two subcategories: (1) performance-approach and (2) performance-avoidance. Individuals with a performance-approach orientation aim to outperform their peers in order to gain recognition (i.e., play to win), while those with a performance-avoidance goal orientation try to avoid losing in order to avoid looking foolish or incompetent (i.e., play not to lose) (Elliot and Harackiewicz, 1996). Performance-avoidance goal orientation has been positively correlated with test anxiety and a reluctance to seek help when needed (Middleton and Midgley, 1997), and it is negatively correlated with learning and academic performance (Elliot and Church, 1997; Payne et al., 2007).

Research on the adaptiveness of a performance-approach orientation is mixed. Some studies suggest that when paired with a mastery goal orientation, performance-approach orientation can be adaptive, but only under certain conditions (e.g., lower fear of failure and uncertainty) (Elliot and Church, 1997; Darnon et al., 2007). More recent research suggests that adolescents, especially girls from high socioeconomic status communities experience overwhelming academic and psychosocial stress due in part to the pressure to out-achieve their peers and to compete for limited privileges (e.g., entry at prestigious universities) (Luthar et al., 2013; Spencer et al., 2018).

In contrast to this emphasis on social comparison among those with a performance goal orientation, those with a mastery goal orientation were motivated by a desire to increase their competence, knowledge, and understanding (Pintrich, 2000). Mastery goal orientation is based in high competency expectancies and intrinsic motivation (Elliot and Church, 1997), and it has been positively associated with effective learning strategies, such as self-regulatory behaviors (Payne et al., 2007) and high levels of self-efficacy and interest (Middleton and Midgley, 1997).

### Purpose and Self-Efficacy

Research has demonstrated that adolescents who disproportionately valued and attained extrinsic goals rather than intrinsic goals (e.g., personal growth, close relationships, community involvement) experienced poorer mental health and a reduced sense of self-efficacy (Liang et al., 2016). These adolescents pay a heavy personal price when their definitions of success tie their self-worth to performance over purpose. In contrast, those who pursue intrinsic goals, such as cultivating a sense of purpose and contributing to the good of others, may be less subject to the pressures of competition and social comparison, and thus enjoy greater health and self-esteem (Lyman and Luthar, 2014; Spencer et al., 2018). Adolescents who pursued purpose over performance described being less driven by fears of failure, and more driven by passions to fulfill a calling. Thus, despite the surrounding achievement pressures, they remained centered and self-assured.

Thus, goal orientation and self-efficacy appear to play a combined role in academic engagement and outcomes (Lent et al., 1996; Ryan and Deci, 2000). More specifically, self-efficacy refers to individuals’ judgments of their ability to organize and enact behaviors required to perform well (Bandura, 1977). In other words, it is an individuals’ sense of whether they are able to perform in the way they hope. It is a dynamic set of self-beliefs that are linked to different areas of functioning in different domains, as one person cannot feel competent at all tasks (Bandura, 1997; Lent, 2012). Thus, an individual may have a strong sense of self-efficacy in one area of performance, such as an academic subject area, but a low sense of self-efficacy in another domain, such as social skills. Adolescents who are highly efficacious may have a wide array of experiences that contribute to deeper, more accurate understandings of their capabilities that inform which behaviors to further pursue. Ultimately, self-efficacy beliefs are thought to lead to corresponding behaviors. For example, a strong sense of self-efficacy regarding one’s academic capabilities is necessary to motivate individuals to engage in behaviors that lead to academic achievement (Jinks and Morgan, 1999).

One explanation for why self-efficacy is tied to behavior is that when adolescents are highly efficacious, they are more likely to persevere in pursuing their ultimate aims or long-term goals. Thus, it is expected that adolescents who are highly efficacious compared to their counterparts may be more likely to pursue long-term goals or ultimate aims that are tied to academic success. For instance, adolescents who are highly efficacious are more likely to complete their education and engage in behaviors that prepare them for a range of career options (Zimmerman, 1990; Bandura et al., 2001). More specifically, self-efficacy beliefs are associated with reduced delinquency behaviors and increased academic grades (Carroll et al., 2009). Ultimately, these beliefs have positive long-term effects on adolescents’ academic success (Hwang et al., 2016), especially in school contexts characterized by a growth mindset (Høigaard et al., 2015). This latter finding is particularly relevant in the current study given that the MPOWER program is designed to create a classroom environment that fosters a sense of BTS purpose rather than just success for the sake of social comparison. MPOWER aims to increase purpose and decrease performance-avoidance goal orientations. Moreover, the program aims to increase self-efficacy beliefs that ultimately lead to increases in academic achievement (Carroll et al., 2009; Høigaard et al., 2015; Hwang et al., 2016).

### MPOWER: a Purpose Program

This school-based program (for more details on MPOWER, see Klein et al., 2019) was designed to guide students in exploring purpose through a weekly, 50-min classroom-based, yearlong curriculum and through supportive relationships such as additional one-to-one mentoring by a guidance counselor and a classroom community of co-participants.

Moreover, MPOWER incorporates the 4 P’s of purpose framework by using the curriculum and these formative relationships to help adolescents identify and cultivate their *passion* by identifying core values and considering whether these core values align with daily activities or pursuits. As adolescents identify their passion(s), they begin reflecting on their *propensity* by identifying their strengths and skills relevant to pursuing their purpose; if adolescents believe they do not possess the propensity, then they identify ways to develop the strengths and skills needed or consider alternative aspirations that may be a better fit for them. Finally, adolescents are also supported to explore potential *prosocial benefits* of their aspirations, and asked to reflect on the impact of their purpose on people’s lives.

With respect to fostering the BTS aspect of purpose, MPOWER uses a number of exercises that helped participants understand how fulfilling their aspirations may impact the world. For example, participants played an adapted version of “The Dictator Game” which has been shown to promote altruistic intentions and prosocial behaviors in youth (Benenson et al., 2007). More specifically, participants completed multiple rounds of the aforementioned game where they can give or receive money with their peers, which is tied to their ranking. The majority of participants exhibited prosocial behaviors by helping their peers at the expense of their own ranking. Upon completion of the activity (and other activities similar to it), participants explore their own desired prosocial impact or contributions they would like to make. The BTS aspect of purpose is emphasized in MPOWER because it was created to improve academic engagement for adolescents with marginalized identities. Familial struggles (e.g., precarious immigration status, low SES) may motivate adolescents to pursue a purpose that helps their family and/or themselves escape adversity associated with their marginalized backgrounds (e.g., poverty) (Liang et al., 2017; Gutowski et al., 2018). Finally, a brief psychological intervention that promoted a self-transcendent purpose (i.e., BTS purpose) improved academic performance over several months (Yeager et al., 2014).

In addition to fostering BTS intentions, participants were asked to engage in personal reflection through journaling and writing exercises which primed them to think about their core values and character strengths (Cohen et al., 2009). Experiential group activities were used as a way of increasing a sense of community and academic performance among the participants (Nagaoka et al., 2013). These activities served as a starting point for participants’ one-to-one mentoring sessions with the MPOWER instructor through which they were given an opportunity to reflect more deeply and share personal experiences that they may have been reluctant to share in the larger context of the classroom. These individual sessions focused on enabling participants to discuss how their lived experiences, families, cultures, and communities informed their long-term aspirations/purpose. Participants were taught the language and framework of purpose, and engaged in introspective reflection in order to help them create a narrative in which their past and present experiences could be connected with their future aspirations (Wilson, 2011).

### Current Study

The current study examined the effectiveness of the MPOWER program for increasing participants’ BTS aspect of purpose, mastery goal orientation, self-efficacy, and academic achievement. We hypothesized that students participating in the MPOWER program would show: (a) increased BTS purpose and academic achievement (i.e., grade-point average); (b) increased mastery goal orientation and self-efficacy; and (c) decreased performance-approach and performance-avoidance goal orientation.

## Materials and Methods

### Study Design and Participants

**FIGURE 1:**
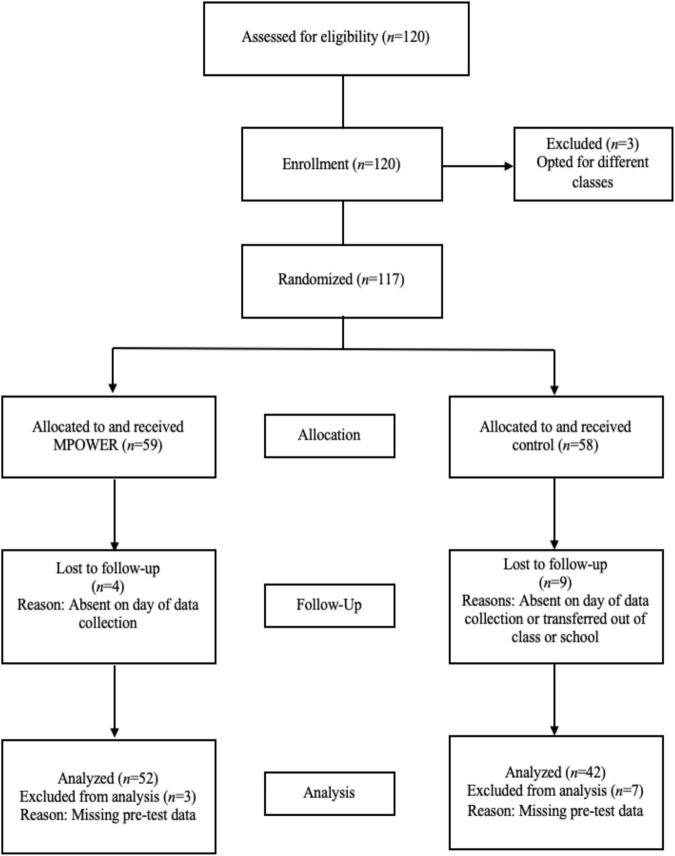
Flow of participants through each stage of the randomized controlled trial.

This study was pre-approved by the Institutional Review Board at Boston College (IRB Protocol: 16.145.02-3) which required pre-approval by the participating school’s principal and district superintendent and pre-registered the measures that were used to assess pre-test and post-test scores. This study focused on a sample of 12th grade students (*N* = 94, control *N* = 42, 61.7% female) from a U.S. public high school in the northeast. As it is the only high school within the district, its students are representative of the district’s diverse working- to middle-class community. Additionally, this sample represented 30% of the schools’ student body in the 12th grade. The MPOWER program was delivered to only seniors. Figure 1 displays how participants were randomly assigned to the program (i.e., MPOWER) and control group (i.e., academic enrichment/study hall class). The sample was predominantly female due to female students exhibiting greater academic performance than male students at this high school. In order to qualify for the study hall period that MPOWER and the academic enrichment class were offered during, students must be on track to graduate and have a free period that can be filled by the elective.

The participants in the control group were given an alternative curriculum that focused on increasing academic engagement and improving academic performance. In addition to giving participants allotted time in school to complete their academic work, they were also provided a curriculum that taught them a variety of non-cognitive skills that are correlated with improved student outcomes. These non-cognitive skills included intrapersonal skills, such as task, organizational, and time management as well as social emotional skills, including stress regulation, impulse control, and capacity for optimism. Moreover, participants in the control were offered group and individual tutoring as needed. The control condition is viewed as “treatment as usual” because this is typically what students receive during this elective period; the MPOWER intervention was allowed to be piloted during the elective period.

The sample included participants who self-identified as White/Euro-American (*N* = 42, 64.2% female), African-American/Black (*N* = 11, 45.4% female), Haitian (*N* = 7, 71.4% female), Caribbean (*N* = 1, 100% female), Latino or Hispanic (*N* = 7, 57.1% female), Brazilian or Portuguese (*N* = 12, 58.3% female), Asian (*N* = 8, 50.0% female), Asian-Americans (*N* = 3, 66.7% female), Pacific Islander (*N* = 1, 100% female), and Native American (*N* = 2, 100% female). Based on parental education level, the sample represented a range of socioeconomic backgrounds (8.5% of parents had a master’s or doctoral degree, 37.2% of parents had an associate’s or bachelor’s degree, 37.2% of parents graduated from high school, 17.0% of parents did not graduate from high school). Participants were given pre-test surveys prior to their respective interventions that assessed their self-efficacy, goal orientation, and purpose; they also completed post-test surveys after their respective interventions (see section “Measures” for more detail).

### Measures

#### Purpose

The Claremont Purpose Scale is designed to assess purpose development in adolescents (Bronk et al., 2018). It includes three subscales with four items each: (1) Purpose Meaningfulness (e.g., “How clear is your sense of purpose in your life?,” four items, α = 0.92); (2) Purpose Engagement (e.g., “How hard are you working to make your long-term aims a reality?,” four items, α = 0.86); (3) BTS Purpose (e.g., “How often do you hope to leave the world better than you found it?,” α = 0.91). Response options range from 1 (strongly disagree) through 5 (strongly agree). Higher average scores indicate a stronger sense of purpose. Construct validity of the scale has been documented with adolescents (Bronk et al., 2018). The reliability coefficient for the current study was α = 0.871 (pre-test) and α = 0.930 (post-test).

#### Goal Orientation

The Patterns of Adaptive Learnings Skills Scale (PALS; Midgley et al., 2000) has been used to assess the goal orientation of students at varying developmental stages, including elementary, middle, and high school students (see Pintrich, 2000; Bong, 2001). It is comprised of three subscales: (1) Mastery Goal Orientation, which consists of five items (e.g., “It’s important to me that I learn a lot of new concepts this year”; α = 0.85); (2) Performance Goal Orientation, which consists of five items (e.g., “It’s important to me that other students in my class think I am good at my class work”; α = 0.89); and (3) Performance-Avoidance Goal Orientation, which consists of four items (e.g., “It’s important to me that I don’t look stupid in class”; α = 0.74). A 5-point response scale was used, ranging from 1 (not at all true) through 5 (very true). Higher average scores within each subscale indicate a stronger goal orientation on that subscale. The reliability coefficient for the current study was α = 0.853 (pre-test) and α = 0.881 (post-test).

#### Self-Efficacy

The New General Self-Efficacy Scale is designed to assess self-efficacy in adolescents (Chen et al., 2001). It is comprised of eight items (e.g., “I will be able to achieve most of the goals that I have set for myself,” α = 0.85 to α = 0.90). Response options range from strongly disagree (1) to strongly agree (5). Higher average scores indicate a higher level of general self-efficacy. The reliability coefficient for the current study was α = 0.871 (pre-test) and α = 0.943 (post-test).

#### Grade Point Average

Annual grade point average (GPA) is an indicator of a Student’s overall academic performance in their classes for the academic school year. We documented students’ GPAs from pre-test and post-test to determine if there were any significant changes in their academic performance.

### Procedures

In anticipation of data collection, student participants’ parents received a co-signed letter from the principal and research team, as well as informed passive consent forms. To deny consent for their child, parents were asked to return the signed letter, email a school administrator, or email a member of the research team.

The survey was administered twice during a single class period, first as a pre-test in September 2017 and then as a post-test in May 2018. The MPOWER instructor and research assistants arrived at each class with internet enabled laptops. The study was described to students, and they provided informed assent. All students whose parents had given passive consent were invited to participate in the study. The MPOWER instructor and a research assistant were present to assist students and answer questions that arose.

### Missing Data

Students with both a pre-test and post-test were included in the final sample. Those who missed either the pre-test (*N* = 10) or post-test (*N* = 13) data collection were excluded from this study. Any other missing data were replaced using an expectation maximization (EM) algorithm to create a single new dataset where the missing values are imputed with maximum likelihood values (Acock, 2005). EM is expected to produce unbiased parameter estimates when the data are missing cases at random (MCAR) (Acock, 1997 as cited in Fox-Wasylyshyn and El-Masri, 2005). Little’s MCAR test (Little, 1988) was conducted to see whether the EM algorithm converged; our analysis suggested that our data was MCAR and converged.

### Data Analytic Strategy

Separate one-way analyses of covariance (ANCOVAs) were conducted to test the effects of the MPOWER program on the outcomes of interest. Four outcome measures (purpose, goal orientation, self-efficacy, GPA) were used with pre-test scores for each outcome included in the analyses as covariates. Covariates were included in these analyses to partial out the effects of pre-test scores. The students in this study were randomly assigned to the intervention and control group; thus, group differences were not included as covariates. ANCOVA tables include the estimated marginal means for all analyses. An alpha of 0.05 was established as the criterion for significant results.

Analyses were conducted using SPSS 28.0, which produces an index of effect sizes using the partial eta-square; however, we calculated omega-square which is considered a less biased alternative with smaller samples (Okada, 2013). Omega-square can be used like Cohen’s *d*, (Cohen, 1988) to assess the effect of the intervention. The metric for assessing omega square for small, medium, and large effect sizes are 0.01, 0.06, and 0.14, respectively (Fields, 2013).

## Results

Table 1 presents the means and standard deviations for all outcome measures at pre-test and post-test for students in the intervention and comparison group. The intercorrelations between pre-test outcomes for students in the intervention and comparison group are presented in Table 2. Purpose was correlated with self-efficacy and modestly correlated with mastery and performance approach goal orientation. Although there were several significant correlations, none of them were higher than *r* = 0.523, suggesting there is some overlap, but that they are measuring different constructs. The results of the ANCOVA analyses for the intervention and control group, along with effect sizes, are presented in Table 3. Findings indicated that there was a significant effect of the program for five outcomes. Specifically, the program had a significant main effect on MPOWER participants’ grade point average, generalized self-efficacy, performance approach goal orientation, performance avoidance goal orientation, and the BTS aspect of purpose. The effect sizes for all main effect sizes range from very small to moderate ω^2^ = −0.007 to 0.080.

**TABLE 1:** Means and standard deviations at pre-test and post-test for intervention and comparison groups.

	MPOWER (*n* = 52)		Control (*n* = 42)	
	Pre-test means (SD)	Post-test means (SD)	Pre-test means (SD)	Post-test means (SD)
Self-efficacy	29.33 (4.92)	32.35 (6.04)	30.26 (3.93)	29.76 (6.32)
Purpose	3.58 (0.64)	3.90 (0.78)	3.44 (0.68)	3.56 (0.79)
Purpose meaningfulness	3.15 (0.97)	3.64 (1.04)	3.17 (0.82)	3.45 (0.91)
Purpose engagement	3.67 (0.76)	3.82 (0.90)	3.48 (0.84)	3.61 (0.82)
Purpose BTS	3.92 (0.77)	4.23 (0.84)	3.67 (0.93)	3.61 (1.02)
Mastery	3.95 (0.66)	3.95 (0.77)	4.00 (0.54)	3.91 (0.84)
Performance-approach	2.70 (1.04)	2.33 (1.09)	2.85 (0.87)	3.08 (0.98)
Performance-avoidance	3.16 (0.97)	2.52 (1.10)	3.11 (0.86)	3.08 (0.97)
Grade point average	2.98 (0.74)	3.04 (0.72)	2.93 (0.77)	2.93 (0.77)

**TABLE 2:** Inter-correlations among participants’ pre-test measures.

Variables	1	2	3	4	5	6	7	8
1. Self-efficacy	–							
2. Purpose	0.523**	–						
3. Purpose meaningfulness	0.482**	0.700**	–					
4. Purpose engagement	0.449**	0.834**	0.344**	–				
5. Purpose BTS	0.284**	0.801**	0.247**	0.637**	–			
6. Mastery	0.105	0.213*	0.040	0.219*	0.246*	–		
7. Performance-approach	0.083	0.252*	0.289*	0.142	0.146	0.027	–	
8. Performance-avoidance	–0.177	0.137	0.118	0.114	0.087	0.049	0.712**	–

*p < 0.05; **p < 0.01.

**TABLE 3:** ANCOVA results among participants’ post-test measures.

Outcomes	MPOWER (*n* = 52)	Control (*n* = 42)	Group partial		
	Mean (SE)	Mean (SE)	*F* ^a^	*p*-value	ω^2^
Purpose	3.84 (0.08)	3.62 (0.09)	3.32	NS	0.012
Purpose meaningfulness	3.65 (0.12)	3.44 (0.13)	1.34	NS	0.002
Purpose engagement	3.76 (0.10)	3.68 (0.11)	0.313	NS	–0.005
Purpose BTS	4.14 (0.10)	3.71 (0.11)	8.44	<0.005	0.042
Self-efficacy^b^	32.57 (0.80)	29.48 (0.89)	6.69	<0.05	0.052
Mastery	3.97 (0.10)	3.90 (0.12)	0.203	NS	–0.007
Performance-approach	2.38 (0.12)	3.03 (0.14)	12.94	<0.001	0.080
Performance-avoidance	2.51 (0.13)	3.09 (0.14)	9.50	<0.005	0.064
Grade point average	3.02 (0.01)	2.96 (0.02)	10.42	<0.005	0.001

*Estimated marginal means are reported; covariates are pre-test outcome measures.*

*^a^ANCOVA assumptions were tested prior to conducting analyses. When a pretest outcome × group interaction term was significant and the slopes were both in the same direction in tests for homogeneity of regression slopes, we reported the F-value associated with the main effect of group.*

*^b^Bootstrapping was utilized for New General Self-Efficacy due to heterogeneity of regression slopes.*

### Purpose

A one-way ANCOVA was conducted to compare the effectiveness of the MPOWER intervention while controlling for pre-test scores, as shown in Table 1. There was not a significant difference in composite purpose post-test scores [*F*_(1, 91)_ = 3.32, *p* > 0.05, ω^2^ = 0.012] between the conditions. *Post hoc* tests showed there was a trend toward a difference in the composite purpose score between the MPOWER and control group, but it was not significant (*p* = 0.071).

Analyses with the purpose measure subscales demonstrated that there was not a significant difference across program and control groups post-test scores for purpose meaningfulness [*F*_(1, 91)_ = 1.34, *p* > 0.05, ω^2^ = 0.002] and purpose engagement [*F*_(1, 91)_ = 0.313, *p* > 0.05, ω^2^ = −0.005]. However, there was a significant difference in post-test BTS purpose [*F*_(1, 91)_ = 8.44, *p* < 0.01, ω^2^ = 0.042]. The estimated marginal means showed that MPOWER students reported significantly higher BTS purpose post-test scores, *M* = 4.14, 95% CI [3.94, 4.34] compared to control students *M* = 3.71, 95% CI [3.49, 3.93].

### Self-Efficacy

A one-way ANCOVA controlling for pre-test scores demonstrated a significant difference in self-efficacy post-test scores [*F*_(1, 91)_ = 6.69, *p* < 0.05, ω^2^ = 0.052]; MPOWER students reported significantly higher self-efficacy post-test scores, *M* = 32.57, 95% CI [31.01, 34.12] than did control students, *M* = 29.48, 95% CI [27.75, 31.22].

### Goal Orientation

#### Mastery

A one-way ANCOVA was conducted to compare the effectiveness of MPOWER while controlling for pre-test scores. There was no significant difference in mastery goal orientation post-test scores [*F*_(1, 91)_ = 0.203, *p* > 0.05, ω^2^ = −0.007] between MPOWER and control students.

#### Performance-Approach

A one-way ANCOVA controlling for pre-test scores demonstrated a significant difference in performance-approach goal orientation post-test scores [*F*_(1, 91)_ = 12.94, *p* < 0.001, ω^2^ = 0.080]. MPOWER students reported significantly lower performance-approach goal orientation post-test scores, *M* = 2.38, 95% CI [2.14, 2.61] than did control students, *M* = 3.03, 95% CI [2.77, 3.30].

#### Performance-Avoidance

A one-way ANCOVA controlling for pre-test scores demonstrated a significant difference in performance-avoidance goal orientation post-test scores [*F*_(1, 91)_ = 9.50, *p* < 0.005, ω^2^ = 0.064]. MPOWER students reported significantly lower performance-avoidance goal orientation post-test scores, *M* = 2.51, 95% CI [2.27, 2.76] than did control students, *M* = 3.09, 95% CI [2.82, 3.37].

### Grade Point Average

A one-way ANCOVA controlling for pre-test GPA demonstrated a significant difference in GPA at post-test [*F*_(1, 91)_ = 10.42, *p* < 0.005, ω^2^ = 0.001]. MPOWER students reported significantly higher GPA post-test scores, *M* = 3.02 than did control students, *M* = 2.96.

## Discussion

The findings demonstrated that MPOWER participants dropped in levels of performance-approach and performance-avoidance goal orientations. That is, by the end of the program they were less concerned with outperforming their peers and avoiding tasks due to fears of looking foolish and/or incompetent. This change in attitudes is notable, given that adolescents typically over focus on comparison with others rather than a genuine interest in learning for their own sake. Previous research has revealed adolescents’ preoccupation with extrinsic motivations, such as peer (Stewart, 2008) and familial (Spencer et al., 2018) expectations when pursuing academic and professional goals. Thus, it appears that MPOWER may cultivate new learning attitudes that lessen the hold of these pressures on participants and enable them to feel less self-conscious as they explore intrinsically meaningful goals. As a result of a decreased performance-avoidance goal orientation, participants in MPOWER may be more comfortable with seeking help (Middleton and Midgley, 1997). Effective help-seeking is beneficial for achieving academic goals (Newman, 2002). Previous research also suggests that adolescents with more adaptive goal orientations may have greater tolerance of uncertainty with respect to achieving academic and professional goals (Darnon et al., 2007). Indeed, it is important for adolescents, especially those with college aspirations, to have the courage to take risks, including engaging in novel activities.

There was not a significant difference between MPOWER and control participants on levels of mastery goal orientation (i.e., a focus on increasing knowledge and competence). Participants across groups may have had similar levels of mastery goal orientation due to factors relevant to normative adolescent development. Erikson (1968) described adolescence (ages 12–18 years old) as a stage where individuals resolve the conflict of identity vs. role confusion; adolescents are focused on becoming independent, adapting and growing into changes that occur during adolescence, and look for a sense of belonging and fit with greater society. Given the desire for a sense of belonging, adolescents are susceptible to external pressures, for example, their parents and peers (Erikson, 1968). As mentioned previously, mastery goal orientation is different from performance goal orientations, as it is not grounded in social comparison. Therefore, performance orientations may be more susceptible to change for MPOWER participants as they are achieving a better grasp of their identity, in contrast to the role confusion that may be experienced by their control counterparts.

Program participants also reported an increase in the BTS aspect of purpose. A primary aim of MPOWER is to increase this type of purpose given that it is associated with various beneficial outcomes, including personal and communal growth (Hill et al., 2010; Sumner et al., 2018) and life satisfaction (Bronk and Finch, 2010). Moreover, Spencer et al. (2018) found that adolescent girls who were more other-focused (i.e., committed to pro-social aims beyond the self) were better able to manage pressures to perform and peer competition. Thus, it is not surprising that participants in MPOWER were less susceptible to external pressures (i.e., performance approach and performance avoidance goal orientations) as they became increasingly committed to contributing to others vs. a sole focus on self-oriented aims.

The lack of significant difference across groups in personal meaningfulness (i.e., having a clear long-term aspiration) and goal engagement (i.e., engaging in activities relevant to one’s purpose) may be explained in that such changes require a period of sustained search for purpose—a state that is associated with greater clarity of purpose (Blattner et al., 2013). It stands to reason that high school students participating in a 1-year program have not yet had adequate time to identify their specific purpose and engage in activities relevant to that purpose. The more striking finding is that after just 1 year, they are intent on making the world around them better (i.e., BTS subscale), even if they have not determined their unique way of doing so (i.e., personal meaningfulness scale).

Indeed, scholars have suggested that the development of one’s sense of purpose parallels Marcia’s (1966) identity development categories: foreclosure, diffusion, moratorium, and achievement (Blattner et al., 2013). For example, a young person may begin by hastily adopting a parent’s/guardian’s ideas about purpose (foreclosure) because it feels safer than having to embark on what can be an overwhelming process of searching for their own unique purpose in life. A person may go through a period of moratorium (high search and low commitment) before achieving purpose (high search and high commitment). When in moratorium, it is conceivable that a person could develop a desire to contribute to the world beyond-the-self long before having a clear idea about how to do this (i.e., before they have clarity on their purpose), and before actively engaging in activities relevant to a clear purpose (which ostensibly would require having a clear purpose first). Because searching for purpose is an inherently stressful stage (Blattner et al., 2013), having a high sense of self-efficacy can be promotive in supporting one through this stage.

Unsurprisingly, students participating in MPOWER reported higher levels of general self-efficacy by the end of the program. It is possible that this increase in self efficacy paired with decreased performance-approach and performance-avoidance orientations, propelled participants to begin exploring the BTS aspect of their purpose. Additionally, when adolescents have high general self-efficacy, an overall belief in their abilities to pursue outcomes and goals (Bandura, 1977), they are more likely to be academically engaged (Schunk and Ertmer, 1999) and successful (Carroll et al., 2009; Hwang et al., 2016). Moreover, strengthening general self-efficacy beliefs among high school students has the potential to improve their self-regulated learning strategies, such as planning, practice, and evaluation (Zimmerman, 1990), as well as their ability to pursue career aspirations (Bandura et al., 2001).

Finally, participation in the MPOWER program was associated with increased GPAs. Existing research suggests that adolescents who have BTS purpose become more committed to academics because they have something worth fighting for Spencer et al. (2018). Moreover, MPOWER participants’ improved self-efficacy and willingness to seek out help from others (i.e., decrease in performance-avoidance goal orientation) may have also contributed to their increased GPAs. Funny, integral shifts in the perception of their own capabilities (i.e., self-efficacy) may also have allowed MPOWER participants to focus more on increasing their competencies and knowledge in order to achieve their purpose aims.

## Strengths, Limitations, and Recommendations

We note several limitations and strengths to our current study. Although the sample was racially diverse, it included students from just one town and one school. Additional research involving more schools and geographical diversity is needed to increase generalizability of findings. Moreover, given our limited sample size, we were not able to include gender, race, socioeconomic status, and ethnicity as covariates in the ANCOVA analyses.

The pre- and post-data were obtained across 1 year; however, longitudinal data over a longer span of time would be useful in determining whether MPOWER has lasting effects beyond participants’ involvement in the program. Additionally, the current study relied on student self-report of purpose, goal orientations, and self-efficacy. Future research may include individual student interviews, as well as utilizing observational methods to triangulate the data more extensively. Finally, there are limitations to using GPA as the sole measure of academic achievement and a reflection of potential academic engagement given the variability in grading criteria amongst different teachers (Lei et al., 2001) and the phenomenon of grade inflation (i.e., instructors lower their standards to improve course ratings by students) (Bejar and Blew, 1981).

Future research would do well to include additional purpose measures, as well as other academic engagement and achievement measures. Future research should utilize larger sample sizes so that covariates of interest, in particular socioeconomic status, can be included. Moreover, it would be helpful to examine the development of BTS purpose alongside other areas of adolescent development, such as moral development, which has been documented to influence civic engagement (Malin et al., 2015). Finally, there may be other factors associated with BTS intentions such as religious affiliation or family make-up (e.g., single parent vs. two-parent households) that future research should examine.

Despite these limitations, this study is one of the few to evaluate a purpose development program for high school students. This study is the first to show that a purpose intervention positively impacts BTS purpose development as well as other academic engagement constructs that can improve academic performance. Moreover, this study provides promising empirical evidence for developing future programs of this nature given that our effect sizes were small to moderate despite our modest sample size.

## Conclusion

The MPOWER program was associated with an increase in the BTS aspect of purpose and self-efficacy among participants. Also, significant changes in performance-approach and -avoidance goal orientations were reported as well as improvement in participants’ GPAs. Especially promising are findings that suggest that participants’ internal attitudes about their own abilities to positively impact their world and susceptibility to external pressures can be changed through participation in MPOWER. Educators and other practitioners should consider creating similar purpose interventions to increase their high school students’ intrinsic motivation and decrease their extrinsic motivation as they pursue their academic goals and develop a BTS purpose. This desire to contribute to the common good appears to be linked to the wellbeing and positive academic outcomes of the students within this study.

## Data Availability Statement

The raw data supporting the conclusions of this article will be made available by the authors, without undue reservation.

## Ethics Statement

The studies involving human participants were reviewed and approved by the Institutional Review Board of Boston College. Written informed consent to participate in this study was provided by the participants’ legal guardian/next of kin.

## Author Contributions

JS designed the study, led the program evaluation, led data analysis, and wrote the majority of manuscript. BrL aided in selecting measures and data collection and wrote several sections of the manuscript. BL provided guidance to JS and BrL and edited the manuscript multiple times, and added several paragraphs to the manuscript. TK the creator of MPOWER, collaborated with JS to think about constructs that he aimed to influence with MPOWER, and also assisted with data collection. AW aided in data collection and analysis and also provided edits and guidance on submission of the manuscript. NH assisted primarily with data analysis, played an integral role in the partnership with high school data was collected at, and identified measures that aided in study. JP provided a school administrator’s perspective on what MPOWER should target and also obtained caretakers’ informed consent and helped with data collection. All authors contributed to the article and approved the submitted version.

## Conflict of Interest

The authors declare that the research was conducted in the absence of any commercial or financial relationships that could be construed as a potential conflict of interest.

## Publisher’s Note

All claims expressed in this article are solely those of the authors and do not necessarily represent those of their affiliated organizations, or those of the publisher, the editors and the reviewers. Any product that may be evaluated in this article, or claim that may be made by its manufacturer, is not guaranteed or endorsed by the publisher.
